# Brain Responses to Letters and Speech Sounds and Their Correlations With Cognitive Skills Related to Reading in Children

**DOI:** 10.3389/fnhum.2018.00304

**Published:** 2018-08-03

**Authors:** Weiyong Xu, Orsolya B. Kolozsvari, Simo P. Monto, Jarmo A. Hämäläinen

**Affiliations:** ^1^Department of Psychology, University of Jyväskylä, Jyväskylä, Finland; ^2^Jyväskylä Centre for Interdisciplinary Brain Research, University of Jyväskylä, Jyväskylä, Finland

**Keywords:** letter-speech sound integration, brain development, magnetoencephalography, auditory cortex, language learning, reading

## Abstract

Letter-speech sound (LSS) integration is crucial for initial stages of reading acquisition. However, the relationship between cortical organization for supporting LSS integration, including unimodal and multimodal processes, and reading skills in early readers remains unclear. In the present study, we measured brain responses to Finnish letters and speech sounds from 29 typically developing Finnish children in a child-friendly audiovisual integration experiment using magnetoencephalography. Brain source activations in response to auditory, visual and audiovisual stimuli as well as audiovisual integration response were correlated with reading skills and cognitive skills predictive of reading development after controlling for the effect of age. Regression analysis showed that from the brain measures, the auditory late response around 400 ms showed the largest association with phonological processing and rapid automatized naming abilities. In addition, audiovisual integration effect was most pronounced in the left and right temporoparietal regions and the activities in several of these temporoparietal regions correlated with reading and writing skills. Our findings indicated the important role of temporoparietal regions in the early phase of learning to read and their unique contribution to reading skills.

## Introduction

Letter-speech sound (LSS) integration is a key step in learning to read for alphabetic languages. The development and reorganization of early readers’ language circuits for supporting automatized LSS integration and how such integration is related to the development of fluent reading are crucial questions from both theoretical and practical point of view (Shankweiler et al., 2008; Dehaene et al., 2015). Research has shown that in early readers, the print-speech convergence (as measured by coactivation in fMRI) in the left reading network (inferior frontal gyrus, inferior parietal cortex, and fusiform gyrus) is a significant predictor of reading achievement measured 2 years later (Preston et al., 2016). In another study using four contrasting languages to find common indices of successful literacy acquisition, highly similar neural organization for print-speech convergence was observed between the languages. Furthermore, such print-speech convergence was suggested as a common brain signature of reading proficiency (Rueckl et al., 2015). However, little is known about the interrelationships between brain mechanisms of speech perception, letter processing, LSS integration and the development of reading skills during childhood.

In order to understand the development of LSS integration, which is a form of audiovisual integration, auditory and visual processes also need to be taken into account. The maturation of auditory and visual cortices is reflected by changes in the auditory and visual evoked responses. In general, the auditory evoked responses have been shown to change greatly with the tendency of shortened latencies and decreased amplitudes from childhood to adulthood (Albrecht et al., 2000). For example, the auditory P1 and N1b (the supratemporal component of the N1) peaks show large age-related decreases in latency. In addition, auditory P1, P1-N1b, and N2 peak amplitudes change throughout childhood with accelerated change around the age of 10 years (Ponton et al., 2000). For the visual components, there is a clear delay in the activation timing in children compared to adults, which progressively increases from occipital (related to low-level visual analysis) to occipitotemporal (related to letters/letter string analysis) and further to temporal areas (related to written word perception) (Parviainen et al., 2006).

It has been shown that audiovisual speech produces audiovisual interaction effects reflected as both suppression of the visual response to lipreading and reduced auditory responses to the speech sound compared with unimodal conditions (Besle et al., 2004, 2008). One study used audiovisual speech and audiovisual non-linguistic stimuli to investigate the developmental pattern of audiovisual interactions in the age range of 5–19 years (Tremblay et al., 2007). The results showed that the strength of audiovisual speech integration significantly correlated with age, whereas the performance on non-speech tasks seemed to be similar across all ages. These findings suggest independent maturational processes for audiovisual speech and non-speech during childhood. Converging evidence from electrophysiological research revealed a systematic relationship between brain responses underlying audiovisual integration (of simple audiovisual sounds and objects) in the time range of the auditory N1 response (about 120 ms) and age between 7 and 16 years (Brandwein et al., 2011). Multisensory processes are thus still developing even in late childhood and this maturation is likely to be reflected in learning and automatization of LSS correspondences, as well as in the associations with reading skill development.

As children learn to read, their sensitivities to print are paralleled by changes in an occipitotemporal negativity N1 (or N170) to words as measured by event-related potentials (Brem et al., 2010; Maurer et al., 2010; Fraga González et al., 2014). This visual N1 has been shown to develop with reading skills, showing an inverted U-shaped developmental trajectory with maximum N1 tuning (selectivity for print) during the second grade and further decrease of the N1 tuning in adults (Maurer et al., 2005, 2006). Neuroimaging studies have localized the visual print-sensitive N1 in a region within the left fusiform gyrus called “visual word form area” (VWFA) (McCandliss et al., 2003; Dehaene and Cohen, 2011). In one recent study (Bach et al., 2013) 19 non-reading kindergarteners were trained in letter-speech sound associations with Graphogame (Lyytinen et al., 2009) for 8 weeks. It was found that the N1 and the VWFA activation in these kindergarteners significantly improved the prediction of reading skills in second grade over behavioral data alone and together with the behavioral measures they explained up to 88% of the variance in reading (Bach et al., 2013). Therefore, visual N1 is considered as a sensitive index of visual letter string processing reflecting important processes for reading fluency (Fraga González et al., 2014, 2017).

Audiovisual integration, which is defined as the interaction between auditory and visual modalities, and its developmental trajectory remain poorly understood. The additive model, which is based on the comparison of multisensory responses to the summed responses from the constituent unisensory conditions [responses to audiovisual stimuli – responses to (auditory stimuli + visual stimuli)], has been frequently used in electrophysiological studies on multisensory integration (Calvert and Thesen, 2004; Stein and Stanford, 2008; Sperdin et al., 2009). Another commonly used approach in audiovisual research is to study the congruency effect (Jones and Callan, 2003; Ojanen et al., 2005; Hein et al., 2007; Rüsseler et al., 2017), which involves a contrast between congruent and incongruent audiovisual pairs. LSS in alphabetic languages consistently activates several language and cross-modal brain regions in adults. Regions particularly in the superior temporal cortices, have been shown in fMRI studies to have heteromodal properties (van Atteveldt et al., 2009). These brain regions have also been implicated in magnetoencephalography (MEG) findings showing LSS sites in the left and right superior temporal sulci (STS) (Raij et al., 2000). Feedback projections from this heteromodal region have also been shown in fMRI studies to modify the response in a modality-specific region of the primary auditory cortex (van Atteveldt et al., 2004). Top-down factors generated by different task demands and instructions also clearly impact multisensory integration (Andersen et al., 2004). For example, use of explicit vs. implicit and passive vs. active experimental task has been shown to influence the brain responses related to LSS (van Atteveldt et al., 2007; Blau et al., 2008).

Accessing the phonological representations for written words and letter strings has been shown to also involve the parietal areas in many studies particularly the supramarginal gyrus (BA 40) and the angular gyrus (BA 39) (Price, 2000; Pugh et al., 2000b; Schlaggar and McCandliss, 2007). Activation in these two posterior regions was found to significantly correlate with cross-modal (auditory and visual) language task performance (Booth et al., 2003). Furthermore, neuroimaging studies have confirmed that activation in the angular gyrus and supramarginal gyrus were associated with phonological (Buchsbaum and D’Esposito, 2008; Sliwinska et al., 2015) and semantic processing (Binder et al., 2009) of written words, respectively. Parietal regions also show differences during phonological processing in children with reading difficulties (Vandermosten et al., 2016). Taken together, there are several temporoparietal brain regions that are suggested to be involved in the process of integrating visual and auditory information for the purpose of reading.

In contrast to the natural relationship between auditory and visual information in audiovisual speech, the association between letters and speech sounds is mostly based upon agreed conventions. Although knowledge of letter-speech sound associations seems easy to acquire within 1 year of reading instruction (Hardy et al., 1972), EEG studies using mismatch negativity (MMN) paradigm (Näätänen, 2001) have found that beginning readers showed protracted development of letter-speech sound associations beyond early school years (Froyen et al., 2009) and such orthographic–phonological integration could serve as a neural signature of successful or failing reading development (Blomert, 2011). Studies on dyslexia have revealed reduced audiovisual integration (indexed by cross modal MMN) which is associated with a more fundamental deficit in the auditory processing of speech sounds leading to reading failure (Blau et al., 2009; Žarić et al., 2014). Therefore, audiovisual integration is considered as an important marker associated with reading fluency and has been shown to facilitate visual specialization (indexed by print sensitive N1 in the VWFA) in learning to read (Fraga González et al., 2016, 2017).

Although LSS integration has been shown to be important for reading development (Blau et al., 2009, 2010; Blomert and Froyen, 2010; Blomert, 2011), reading is also dependent on other cognitive skills. Several behavioral measures such as phonological awareness, verbal short-term memory and rapid automatized naming (RAN) have been shown to be closely associated with reading skills and provide a good estimation of risk for dyslexia (Pennington and Lefly, 2001; Puolakanaho et al., 2007; Melby-Lervåg et al., 2012). These cognitive measures have been shown to be important mediators of the prediction of reading outcome from brain responses as measured by ERPs (Lohvansuu et al., 2018).

In the present study, we measured auditory responses to speech and visual responses to letters as well as audiovisual integration related responses of letter-speech sound combinations with MEG with the purpose of linking these brain responses to reading development. Previous studies (Froyen et al., 2008, 2009; Blomert, 2011) have often used an audiovisual oddball design and shown a long developmental trajectory for LSS integration. We used an experimental design with equal numbers of unimodal and bimodal stimuli as well as equal numbers of congruent and incongruent audiovisual stimuli. This allows a more direct examination of the LSS integration as well as separating the unimodal effects from the audiovisual effects. We used regression-based methods (controlling for age) to explore the relationship between the neural-level responses to speech sounds, visual letters, audiovisual combinations and behavioral cognitive skills. We expected to see associations between responses to the speech sounds and phonological and reading skills (e.g., Lohvansuu et al., 2018), between the visual N1 and reading skills (e.g., Brem et al., 2010; Maurer et al., 2010; Fraga González et al., 2014), and importantly between the brain measures of LSS integration and reading skills (Blau et al., 2009; Blomert and Willems, 2010; Blomert, 2011; Preston et al., 2016; Fraga González et al., 2017).

## Materials and Methods

### Participants

All participants were Finnish speaking school children (6–11 years) recruited through the National Registry of Finland. None of the participants had neurological disorders or problems caused by permanent head injuries, ADHD, delay in language development or language-specific disorders or medication affecting the central nervous system. In total, 32 Finnish children participated in the experiments. Of those three were excluded for the following reasons: two subjects due to excessive head movements and one subject due to low head position in the MEG helmet. The data included in the present study consisted of 29 children (mean age 8.17 years, SD: 1.05 years; 19 girls, 10 boys; 1 left-handed). All participants included had normal hearing as tested with an audiometry and normal or corrected-to-normal vision. This study was carried out in accordance with the recommendations of the Ethics Committee of the University of Jyväskylä. The protocol was approved by the Ethics Committee of the University of Jyväskylä. All children and their parents were informed about the project and they gave written consent in accordance with the Declaration of Helsinki to participate in the study. All subjects received gifts (movie tickets or shopping vouchers) as compensation for participation.

### Stimuli and Task

**FIGURE 1:**
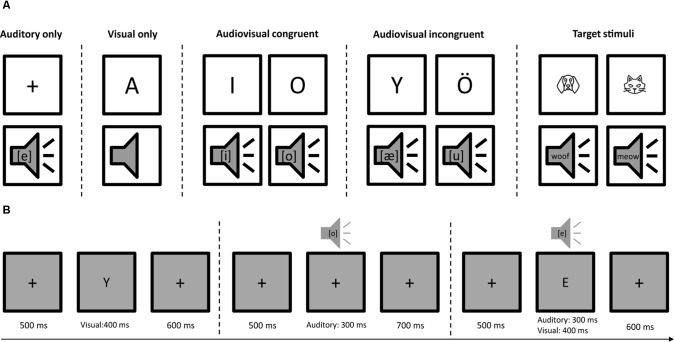
Audiovisual letter-speech sound task. **(A)** Stimuli consisted of auditory only (A), visual only (V), audiovisual congruent (AVC), and audiovisual incongruent (AVI) conditions using eight Finnish letters and their corresponding phonemes. Eight animal drawings and/or animal sounds occurred with 10% probability to keep participants’ attention equally on both auditory and visual stimuli. They were instructed to press a button using their right hand when they saw and/or heard an animal. **(B)** The four types of stimuli (A, V, AVC, and AVI) were presented randomly. Each trial started with 500 ms fixation, followed by auditory (300 ms) and/or visual stimuli (400 ms). The total length of each trial was 1500 ms.

The stimuli consisted of eight Finnish capital letters (A, E, I, O, U, Y, Ä, and Ö) written with Calibri font in black color and their corresponding speech sounds ([a], [e], [i], [o], [u], [y], [æ], and [ø]). Four categories of stimuli, auditory only (A), visual only (V), audiovisual congruent (AVC), and audiovisual incongruent (AVI) were presented in random order with 112 trials for each type of stimuli. The experiment was ca. 20 min in total with two short breaks. The duration of the auditory stimuli was 300 ms. The duration of the visual stimuli was 400 ms. For the audiovisual trials, the auditory and visual stimuli started at the same time. Each trial lasted 1500 ms and started with a fixation cross at the center of the screen for 500 ms, then followed by the presentation of auditory, visual or audiovisual stimuli (**Figure 1**). The visual stimuli were projected on the center of the screen in a gray background. The size of the visual stimuli was 0.6 cm × 0.6 cm for the fixation cross and 2 cm × 2 cm for the letters on a screen 1 m away from the participants. The sounds were delivered through insert earphones using MEG compatible lo-fi sound system at a comfortable loudness level. The stimuli were presented with Presentation (Neurobehavioral Systems, Inc., Albany, CA, United States) software running on a Windows computer. The experiment was conducted in a child-friendly environment in which we told a story of a cartoon character’s adventure in a forest. In order to keep their attention equally on both auditory and visual stimuli, the participants were instructed to press a button using their right hand when they saw an animal drawing or heard an animal sound. In total eight animal drawings and their corresponding sounds were used as target stimuli and they occurred with 10% probability. Feedback (hit or miss) was given immediately after button press.

### MEG and MRI

306-channel MEG data were recorded in a magnetically shielded room using Elekta Neuromag^®^ TRIUX^TM^ system (Elekta AB, Stockholm, Sweden) with 1000 Hz sampling rate and 0.1–330 Hz band-pass filter. The head position in relation to the sensors in the helmet was monitored continuously with five digitized head position indicator (HPI) coils attached to the scalp. Three HPI coils were placed on the forehead and one behind each ear. The position of HPI coils was determined in relation to three anatomic landmarks (nasion, left and right preauricular points) using the Polhemus Isotrak digital tracker system (Polhemus, Colchester, VT, United States) at the beginning of the recording. To allow the co-registration with individual magnetic resonance images (MRIs), an additional set of scalp points (>100) randomly distributed over the skull were also digitized. Electrooculogram (EOG) was recorded with two electrodes attached diagonally slightly below the left and slightly above the right eye and one ground electrode attached to the collarbone. The MEG was recorded in 68° upright gantry position.

Individual structural MR images were acquired from a private company offering MRI services (Synlab Jyväskylä). T1-weighted 3D-SE images were collected on a GE 1.5 T (GoldSeal Signa HDxt) MRI scanner using a standard head coil and with the following parameters: TR/TE = 540/10 ms, flip angle = 90°, matrix size = 256 × 256, slice thickness = 1.2 mm, sagittal orientation.

### Behavioral Assessment

Cognitive skills were tested on a separate visit. The behavioral tests included the following: Wechsler Intelligence Scales for Children Third edition (Wechsler, 1991) and Wechsler Preschool and Primary Scales of Intelligence (Wechsler, 2003) for children above 6 years and for 6-year-olds, respectively. Block design (visuospatial reasoning), vocabulary (expressive vocabulary), and digit span (forward and backward; working memory) subtests were administered. In the block design test, the children are shown how to arrange blocks with red and white color to form a design and they have to build the same design. In more difficult sections the children are only shown the design in a figure and they have to build it. In the vocabulary test, the children hear a word and they have to describe the meaning of that word. In the digit span test, series of numbers are said to the participant and they have to repeat them either in forward or backward order. These tests were used to assess the children general cognitive skills and used as control variables for the possible associations between phonology and reading measures and the MEG indices.

Phonological awareness was tested using the Phonological processing task from NEPSY II (Korkman et al., 2007). In this task, the child is first asked to repeat a word and then to create a new word by leaving out a syllable or a phoneme, or by replacing one phoneme in the word with another phoneme.

Rapid automatized naming (Denckla and Rudel, 1976), in which pictures of five common objects or five letters had to be named as quickly and as accurately as possible. The objects and letters were arranged in five rows each containing 15 objects. The task was audio-recorded and the time in seconds was calculated from the recording to be used in the analyses.

Three reading tests were included: word list reading using standardized test of word list reading (Häyrinen et al., 1999), number of correctly read words in 45 s was used as the score; non-word list reading based on Tests of Word Reading Efficiency (Torgesen et al., 1999), number of correctly read non-words in 45 s was used as the score; pseudoword text reading (Eklund et al., 2015), number of correctly read words and total reading time were used as the scores. Writing to dictation was also assessed in which the child heard 20 words and had to write them on a sheet of paper. Number of correctly written words was used as the score.

### Data Analysis

Data were first processed with Maxfilter 3.0^TM^ (Elekta AB) to remove external interference and correct for head movements. Bad channels were identified manually and were excluded and later reconstructed in Maxfilter. The temporal extension of the signal-space separation method (tSSS) was used in buffers of 30 s (Taulu et al., 2004; Taulu and Kajola, 2005; Taulu and Simola, 2006). Head position was estimated in 200 ms time windows and a 10 ms step for movement compensation. The MEG data were transformed to the mean head position across the recording session.

Data were then analyzed using MNE Python (0.15) (Gramfort et al., 2013). First, continuous MEG recordings were low-pass filtered at 40 Hz and epoched into -200 to 1000 ms trials relative to the stimulus onset. Data were then manually checked to remove any head movement-related artifacts and electronic jump artifacts. Then independent component analysis (ICA) using fastICA algorithm (Hyvärinen and Oja, 2000) was applied to remove eye blinks, horizontal eye movements and cardiac artifacts. MEG epochs exceeding 1 pT/cm for gradiometer or 3 pT for magnetometer peak-to-peak amplitudes were excluded from further analysis. Event-related fields were obtained by averaging trials in the four conditions separately. Sum of the auditory and visual response (A + V) was calculated by first equalizing the number of epochs between the unimodal conditions and then adding up the event-related fields of the auditory and visual only conditions. To match the noise level of A + V and AVC conditions and therefore to make these two conditions comparable, a subset of AVC trials was created by randomly selecting half the number of trials from the AVC condition which equates to the noise level in A + V condition.

Individual MRI were processed in Freesurfer (RRID: SCR_001847, Martinos Center for Biomedical Imaging, Charlestown, MA, United States) to obtain the cortical surface for source modeling. Three participants’ MRIs were replaced by age and gender matched MRIs of other children (MRIs were not available for two children and the third one had a bad quality cortical surface reconstruction). Freesurfer reconstructed cortical surface was decimated to about 4098 evenly distributed vertices per hemisphere with 4.9 mm spacing. Cortically-constrained and depth-weighted (*p* = 0.8) L2 minimum-norm estimate (wMNE) (Hämäläinen and Ilmoniemi, 1994; Lin et al., 2006) was calculated using one layer boundary element model (BEM) from the inner skull surface for all current dipoles with a loose orientation of 0.2. The noise covariance matrix was estimated from the raw 200 ms pre-stimulus baseline data over all conditions. For each current dipole, the estimated source amplitudes were calculated by taking the norm of the vectors. Source amplitudes were averaged within each label for the 68 brain regions defined by the Desikan-Killiany Atlas (Desikan et al., 2006). In order to capture the full extent of the sensory event-related field, the auditory source region was defined by a combination of superior temporal and transverse temporal brain areas and the visual source region was defined by a combination of lateral occipital, cuneus, pericalcarine and lingual brain areas. In addition, the fusiform area was defined as a region of interest for the N170 component based on previous studies (Cohen et al., 2000; Dehaene and Cohen, 2011).

In total, five auditory and visual event-related fields, the auditory N1m, N2m and late component, and the visual P1m and N170m were investigated in the present study. Peak latencies of these sensory responses were identified at sensor level (magnetometer) from the grand average of auditory and visual only conditions. The peak latencies were 109 ms (left) and 105 ms (right) for the auditory N1m, 241 ms (left) and 247 ms (right) for the auditory N2m and 448 ms (left) and 463 ms (right) for the auditory late component. The peak latencies were 104 ms (left) and 97 ms (right) for the visual P1m and 204 ms (left) and 192 ms (right) for the visual N170m. For all the four conditions (A, V, AVC, and AVI), the source level brain activities in the auditory or visual source regions were extracted by taking the average source activities of 50 ms time window centered around the peak latencies which were identified in the previous step. For auditory late component, a longer time window of 100 ms was used due to the extended time course of the response. In addition, individual peak latencies for each participant were also detected within the time window of each component in the source space.

### Statistical Analysis

First, partial correlations (controlling for age in months) were calculated between the cognitive skill measures (see above) and the mean amplitudes and peak latencies of brain sensory responses in the four conditions using SPSS Statistics 24 software package (IBM Corp., Armonk, NY, United States). For the integration (A + V–AVC) and congruency (AVC–AVI) comparison, individual source waveforms in 68 brain regions extracted according to Desikan-Killiany atlas was used in nonparametric permutation (Maris and Oostenveld, 2007) *t*-tests with temporal clustering implemented in Mass Univariate ERP Toolbox (Groppe et al., 2011). The time window was selected from 0 to 1000 ms after stimulus onset and the number of permutations was 2000. The cluster alpha was 0.05 for both integration and congruency comparison. The family-wise *p* values were corrected for multiple comparisons. For regions that showed significant (*p* < 0.05) integration or congruency effects, partial correlations (controlling for age in months) were calculated between cognitive skills and brain responses in each of these regions averaged in the time window of the significant clusters. In addition to the source amplitude values, a laterality index [(left-right)/(left+right)] was calculated for the activity from the fusiform gyrus to examine differences in the development of the hemispheric specialization to print as shown for example by (Maurer et al., 2008, 2010).

In addition, linear regression analyses were performed with cognitive skills as the dependent variable in SPSS Statistics 24. Children’s age was entered first into the model followed by the brain responses as independent variables in order to determine if the different brain responses explain independent or overlapping portions of variance in the cognitive skills. Dependent and independent variables were selected based on significant partial correlations.

## Results

### Cognitive Skills and Behavioral Performance

Descriptive statistics of the participants’ cognitive skill measures and their behavioral performance in the cover task during MEG experiment are presented in **Table 1**.

**Table 1:** Descriptive statistics of the participants’ cognitive skill measures and behavioral performance in the cover task during MEG experiment (*N* = 29).

	Mean	*SD*	Range
Age (years)	8.17	1.05	6–11
Block design, raw score	25.53	9.50	10–52
Vocabulary, raw score	28.17	8.71	12–50
Digit span, raw score	11.67	1.88	8–16
NEPSY phonological processing, raw score	42.53	5.74	30–52
Rapid automatic naming(letters), time (s)	40.01	11.20	26–69
Rapid automatic naming(objects), time (s)	60.61	12.63	38–90
Word list reading, number of correct items	58.46	26.32	10–104
Non-word list reading, number of correct items	31.39	14.23	7–67
Non-word text reading, time (s)	118.32	74.74	36–390
Non-word text reading, number of correct words	29.96	5.33	16–37
Writing to dictation, number of correct words	34.07	8.14	10–40
MEG cover task, accuracy, %	96.91	3.85	81–100
MEG cover task, reaction time, ms	642	127	475–1064


### Grand Averages

**FIGURE 2:**
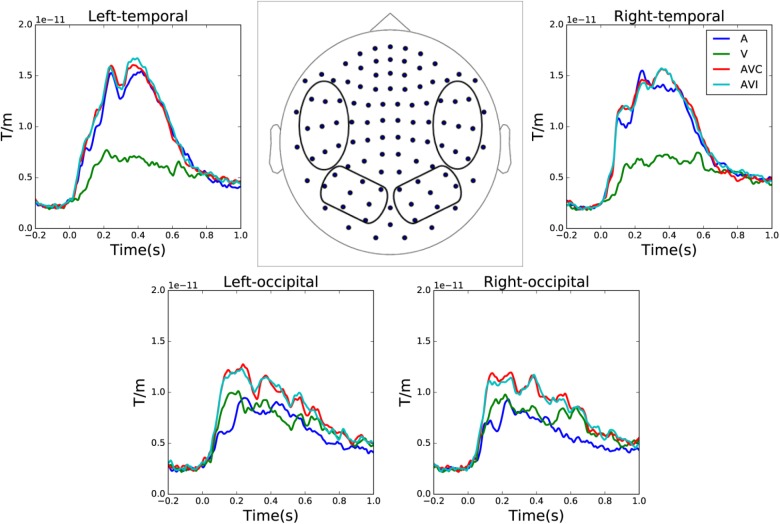
Sensor level grand average of the auditory only, visual only, audiovisual congruent and audiovisual incongruent conditions over left and right temporal and occipital channels. The waveforms are averaged over combined gradiometer channels calculated by vector sum of the two orthogonal gradiometer pairs.

Grand averages of combined gradiometer channels in auditory only, visual only, audiovisual congruent and audiovisual incongruent conditions are shown in **Figure 2**. The waveforms were averaged over left and right temporal and occipital gradiometer channels (within the four circles shown in the sensor layout map).

**FIGURE 3:**
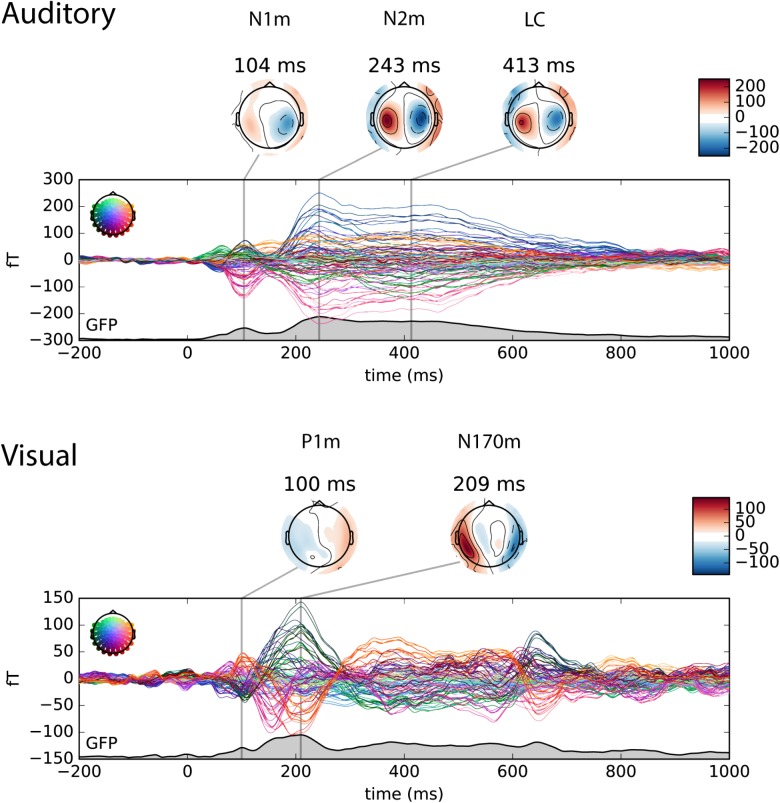
Butterfly plot of the magnetometer channels in the auditory only (upper panel) and the visual only (lower panel) conditions. The individual waveforms are colored by its position in the sensor array as shown on the upper left in each panel. The topography plot shows the auditory N1m, N2m and late component (LC) in the auditory only condition and the visual P1m and N170m in the visual only condition at the local maximum of the global field power (GFP).

The auditory and visual responses were identified in the magnetometer channels based on their topographies and timings. For the visualization purpose, the topography plot of auditory N1m, N2m and late component, and visual P1m and N170m are shown at the local maximum of the global field power (GFP) in **Figure 3**.

### Correlations Between Cognitive Skills and Sensory Brain Responses

No significant correlations were found between the scores in the cognitive skill measures for visuo-spatial reasoning (block design), general verbal skills (vocabulary), or verbal working memory (digit span) and the sensory brain responses. No significant correlations were found between age and the sensory brain responses.

Consistent correlations were found between the phonological processing accuracy, rapid naming speed of letters and auditory N1m, N2m, and LC responses (see **Tables 2–4**). No consistent correlation patterns were observed between peak latencies and cognitive skills (see Supplementary Material). In addition, the left auditory cortex activity at the late time window in response to the audiovisual stimuli showed rather systematic associations with phonology, rapid naming of letters and objects as well as non-word list reading accuracy. N170m amplitude in the left fusiform gyrus in the audiovisual conditions (both AVI and AVC) were significantly correlated with phonological processing. A similar correlation pattern was observed for the auditory only, audiovisual congruent and incongruent conditions in relation to cognitive skills thus indicating a high overlap between these brain measures. In general, we found that the larger the brain response the better the performance in the behavioral tasks for all of the correlations.

**Table 2:** Partial correlations (controlling for age) between cognitive skills and the auditory responses in the auditory only condition.

Auditory	Phonological processing	RAN letters	RAN objects	Word list reading	Non-word list reading	Non-word text time	Non-word text accuracy	Writing to dictation
L, N1m	0.363	-0.219	-0.173	-0.017	0.078	-0.037	0.143	0.141
R, N1m	**0.384^∗^**	-0.312	-0.368	0.304	0.375	-0.226	0.305	0.286
L, N2m	**0.454^∗^**	-0.349	-0.189	0.203	0.323	-0.216	0.312	0.142
R, N2m	0.286	-0.228	-0.265	0.279	0.316	-0.204	0.301	0.273
L, LC	**0.499^∗∗^**	-**0.399^∗^**	-0.358	0.215	0.308	-0.181	0.241	0.234
R, LC	**0.472^∗^**	-0.338	-0.280	0.187	0.258	-0.164	0.284	0.313


*Note: ^∗^*p* < 0.05, ^∗∗^*p* < 0.01. The auditory components are left (L) and right (R) auditory N1m, N2m and late component (LC).*

**Table 3:** Partial correlations (controlling for age) between cognitive skills and the visual responses in the visual only condition.

Auditory	Phonological processing	RAN letters	RAN objects	Word list reading	Non-word list reading	Non-word text time	Non-word text accuracy	Writing to dictation
L, VC, P1	0.343	-0.171	-0.005	-0.241	-0.206	0.255	-0.053	-0.015
R, VC, P1	0.372	-0.212	-0.116	-0.136	-0.081	0.195	0.093	0.075
L, FG, N170	0.282	-0.131	0.036	-0.078	0.011	0.023	0.113	0.027
R, FG, N170	0.218	-0.152	-0.150	-0.080	-0.029	0.079	0.094	0.074
V, LI	0.074	0.062	0.211	-0.017	0.004	-0.125	-0.103	-0.058


*Note: VC, visual cortices; FG, fusiform gyrus; LI, laterality index. The visual responses are left (L) and right (R) visual P1m in the visual cortices and N170m in the fusiform gyrus.*

**Table 4:** Partial correlations (controlling for age) between cognitive skills and the auditory and visual responses in the audiovisual conditions [the first row in each cell is audiovisual congruent (AVC) and the second row audiovisual incongruent (AVI)].

Auditory	Phonological processing	RAN letters	RAN objects	Word list reading	Non-word list reading	Non-word text time	Non-word text accuracy	Writing to dictation
L, AC, N1	0.272	-0.162	-0.124	-0.074	-0.047	0.091	0.098	0.154
	**0.422^∗^**	-0.250	-0.215	-0.025	0.027	-0.014	0.106	0.182
R, AC, N1	0.351	-0.266	-0.315	0.299	**0.390^∗^**	-0.213	0.320	0.257
	0.337	-0.253	-0.352	0.267	0.337	-0.180	0.314	0.253
L, AC, N2	**0.420^∗^**	-0.311	-0.265	0.245	0.378	-0.277	0.229	0.119
	0.338	-0.290	-0.250	0.284	0.346	-0.280	0.235	0.054
R, AC, N2	0.278	-0.247	-0.266	0.290	0.329	-0.230	0.262	0.252
	0.235	-0.203	-0.241	0.216	0.305	-0.198	0.272	0.259
L, AC, LC	**0.506^∗∗^**	**-0.412^∗^**	**-0.405^∗^**	0.311	**0.395^∗^**	-0.288	0.252	0.272
	**0.441^∗^**	**-0.381^∗^**	**-0.394^∗^**	0.218	0.293	-0.251	0.153	0.227
R, AC, LC	**0.448^∗^**	-0.312	-0.235	0.107	0.126	-0.122	0.193	0.267
	**0.456^∗^**	-0.309	-0.241	0.151	0.201	-0.158	0.255	0.293
L, VC, P1	0.292	-0.135	0.010	-0.198	-0.149	0.188	-0.062	0.004
	0.243	-0.087	0.067	-0.308	-0.236	0.281	-0.084	-0.110
R, VC, P1	0.315	-0.197	-0.145	0.015	0.066	0.001	0.057	0.127
	0.297	-0.210	-0.145	-0.024	0.015	0.031	0.091	0.112
L, FG, N170	**0.404^∗^**	-0.261	-0.090	0.014	0.124	-0.011	0.202	0.088
	**0.427^∗^**	-0.280	-0.113	0.064	0.203	-0.025	0.255	0.122
R, FG, N170	0.213	-0.133	-0.173	0.020	0.072	-0.046	0.121	0.191
	0.221	-0.165	-0.235	0.032	0.080	-0.036	0.113	0.170
AV, LI	0.208	-0.079	0.169	-0.050	-0.052	0.045	-0.065	-0.160
	0.220	-0.093	0.239	-0.058	-0.034	0.043	0.010	-0.076


*Note: AC, auditory cortices; VC, visual cortices; FG, fusiform gyrus; LI, laterality index. ^∗^*p* < 0.05, ^∗∗^*p* < 0.01. The auditory components are left (L) and right (R) auditory N1m, N2m and late components. The visual components are left (L) and right (R) visual P1m in the visual cortices and N170m in the fusiform gyrus.*

(Kushnerenko et al., 2002), there is still substantial variation in the response amplitude at school-age that is systematically linked with cognitive skills related to language processing.

In the next step, linear regressions were used to predict the phonological and rapid naming (the dependent variable) using age and the brain responses that showed significant partial correlations as predictors (independent variables). Age was entered first into the model followed first by the significant auditory variables and visual variables using stepwise method and finally by the significant audiovisual variables also using the stepwise method. This model was used to disentangle possible overlapping variance explained by auditory/visual and audiovisual brain responses. In the multiple regression model, as shown in **Table 5** the auditory late component from the left hemisphere was the only significant predictor of the phonological skills and RAN letters.

**Table 5:** Linear regression analysis using phonological and rapid naming as the dependent variable, age was entered first in the model, then the brain responses that showed significant partial correlations as predictors (independent variables).

Cognitive skills	Step	Standardized beta	Δ R2
Phonological processing	1. age	0.256	0.091
	2. Left auditory LC	**0.487^∗∗^**	**0.235^∗∗^**
	3. AVC/AVI responses	ns	ns
RAN letters	1. age	-0.059	0.009
	2. Left auditory LC	-**0.405^∗^**	**0.163^∗^**
	3. AVC/AVI responses	ns	ns


*Note: Beta = standardized Beta coefficient; R2 change = unique variance accounted for at each step of the 2-step (enter method for age; stepwise for brain measures) multiple regression analyses. ^∗^*p* < 0.05, ^∗∗^*p* < 0.01.*

**FIGURE 4:**
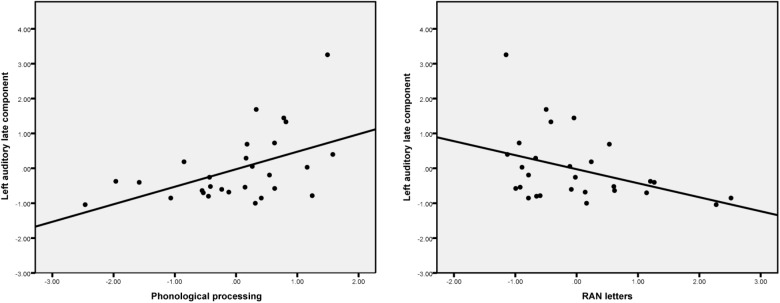
Scatter plots show the partial correlation (controlling for age) between phonological processing skills, RAN letters and the left auditory late component amplitude.

The scatterplots (**Figure 4**) show that, in general, the larger the source activity in the auditory cortex the more likely it is that the child has better phonological processing skills and faster rapid naming abilities.

### Integration and Congruency Effects

#### Integration Effect (A + V vs. AVC)

**FIGURE 5:**
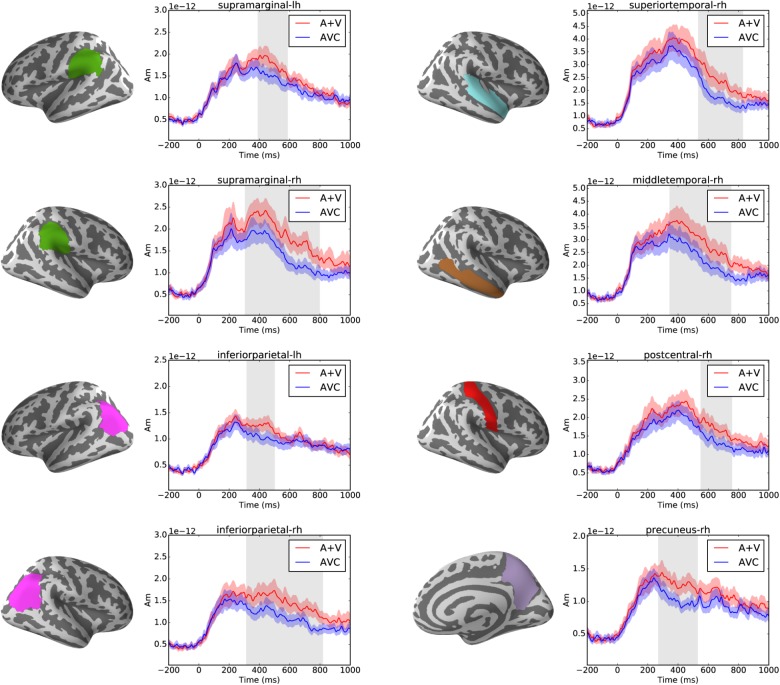
Left panels show the brain regions (as defined in the Desikan-Killiany atlas) that showed significant suppressive integration (A+V–AVC) effects. Right panels show the average source waveform (MNE estimate) extracted from the brain regions with significant clusters. The red and blue shading represent the standard error of the mean and the gray shadings show the time window of the significant cluster.

Cluster-based permutation tests showed that audiovisual integration effect was found in multiple brain regions in the parietal and temporal areas after ca. 250 ms (*p* < 0.05) as shown in **Figure 5**. In total eight significant clusters were found in eight brain regions of the Desikan-Killiany atlas. These clusters were in the left (317–499 ms) and right (315–818 ms) inferior parietal, left (391–585 ms) and right (306–797 ms) supramarginal, right (271–529 ms) precuneus, right (551–755 ms) postcentral and right superior (535–827 ms), and middle (346–749 ms) temporal cortices.

#### Congruency Effect (AVC vs. AVI)

Cluster-based permutation test did not reveal significant effects (*p* > 0.05) in congruency comparison.

### Correlations Between Cognitive Skills and the Brain Activity Related to Multimodal Integration

**FIGURE 6:**
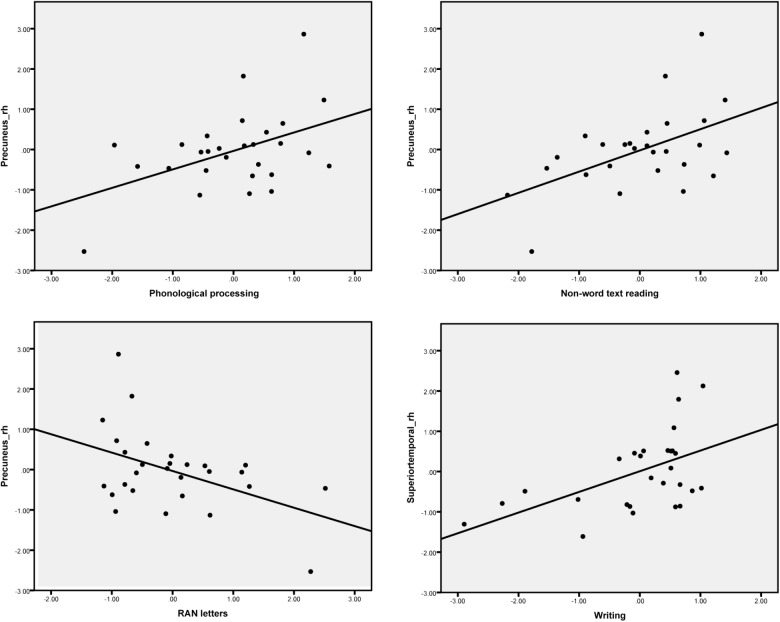
Scatter plots show the most pronounced four partial correlations (controlling for age) between the suppressive effect in the MEG responses for letter-speech sound integration and performance in the cognitive skill tests.

The difference between the AVC and A + V conditions was calculated and the average source amplitudes from the different brain regions in the time window identified by the permutation test were used for the correlation analyses with cognitive skills (**Table 6**). Representative partial correlations between suppressive integration and behavioral tests are shown in **Figure 6**.

**Table 6:** Partial correlations (controlling for age) between cognitive skills and averages of the brain responses in the regions and time windows where significant audiovisual integration effects were revealed by the cluster-based permutation analyses.

A+V–AVC	Phonological processing	RAN letters	RAN objects	Word list reading	Non-word list reading	Non-word text time	Non-word text accuracy	Writing to dictation
Supramarginal_lh	0.365	**-0.421^∗^**	-0.167	0.163	0.233	-0.130	0.297	0.006
Supramarginal_rh	0.324	-0.262	-0.067	0.099	0.096	0.079	0.300	0.293
Inferiorparietal_lh	-0.055	-0.056	0.097	-0.125	-0.036	-0.109	-0.166	-0.136
Inferiorparietal_rh	**0.401^∗^**	-0.353	-0.263	0.192	0.269	-0.126	0.266	0.328
Superiortemporal_rh	0.229	-0.196	-0.259	0.277	0.304	-0.135	0.211	**0.504^∗∗^**
Middletemporal_rh	0.304	-0.215	-0.258	0.306	0.371	-0.230	0.210	**0.408^∗^**
Postcentral_rh	0.215	-0.131	0.104	-0.007	0.133	0.031	0.262	0.263
Precuneus_rh	**0.457^∗^**	**-0.455^∗^**	-0.317	**0.435^∗^**	**0.405^∗^**	-0.186	**0.519^∗∗^**	0.376


*Note: ^∗^*p* < 0.05, ^∗∗^*p* < 0.01.*

## Discussion

In this study, auditory and visual responses, as well as audiovisual integration of letters and speech sounds were correlated with children’s behavioral cognitive skills. Results from the current study revealed that auditory processing, especially the auditory processing in the late time window was the driving force for the correlation between sensory evoked fields and phonological skills. The visual N170 in the left fusiform gyrus in the audiovisual condition was also correlated with phonological skills. In addition, audiovisual suppressive integration was localized mainly in the temporoparietal brain regions and showed an independent contribution from the sensory evoked fields to the reading skills.

It has been shown that the sequence of activation in response to speech sounds is strikingly different in children compared with adults (Wunderlich et al., 2006; Parviainen et al., 2011). Children showed prolonged responses to sound with a major peak at 250 ms in both left and right hemispheres (Parviainen et al., 2011) while a corresponding effect occurred about 100 ms specifically in the left hemisphere in adults (Parviainen et al., 2005). This matches with the current findings that showed a major negative going peak around 250 ms after speech sound onset. The response at 250 ms is usually followed by a second activity peak around 400 ms (Ceponiene et al., 2001, 2005, 2008).

The auditory late component seems to be sensitive to the speech sounds as can be seen from the study in children in which a strong late activation around 400 ms was observed in speech sounds compared to other types of sounds (Parviainen et al., 2011). The activity during the late component time window has been suggested in other studies to be related to late stages of phonological processing (Stevens et al., 2013; Bann and Herdman, 2016) or to orthographic-phonological mapping (Weber-Fox et al., 2003). However, in our study the late processing (around 413 ms) seemed to be linked to the auditory stimuli. This fits with previous studies suggesting that this time window could reflect the effect of speech sound representations (Szymanski et al., 1999; Ceponiene et al., 2001, 2005; Kuuluvainen et al., 2016) and it is sensitive to phonological priming (Bonte and Blomert, 2004). The response has also been suggested to be important for receptive language processing (Ceponiene et al., 2008) which also matched with the correlation pattern of the current study. Overall this could imply that the later stages of integrative speech sound processing are important also for learning to read and for phonological skills. Although the activity around 400 ms seems to mature early in development In the current study, we found correlations between N1, N2, late component and phonological processing for both auditory and AV conditions. Although the regression analysis showed that only the left auditory late component explains unique variance among the brain measures implicating that the early responses do not have independent variance from the late activity that is related to phonological processing. From the time window around 100 ms correlations have been found between brain responses and preschool cognitive skills also in other studies. For example, auditory P1 response has been shown in typically developed children to be associated with phonological and pre-reading skills (Kuuluvainen et al., 2016). In addition, for children at risk for dyslexia, their P1 response amplitudes elicited by speech sound stimuli were smaller compared to controls (Lovio et al., 2010). Similarly, in a study (Hämäläinen et al., 2015) investigating the event-related potentials to tones in children with multiple risk factors for dyslexia, the amplitudes at the P1–N2 time window was correlated with letter knowledge and phonological skills. The N1 and N2 time window has also been shown to be sensitive to reading level differences in response to phonological priming (Bonte, 2004) and nonspeech stimuli (Espy et al., 2004).

The N2 response has been linked to reading and reading-related skills in previous studies. For example, the N2m has been found to correlate with reading skills in children (Parviainen et al., 2011) and the N2 response has been reported to have larger amplitudes in response to speech and non-speech sounds in dyslexic children compared with control group and such enhanced brain responses were correlated with reading skills (Hämäläinen et al., 2013). Furthermore, the brain activity at the N2 time window has been found to correlate with phonological skills, as well as reading and writing accuracy in children with dyslexia (Lohvansuu et al., 2014). The N2m response strength in the left hemisphere in the current study was correlated with phonological skills further supporting the hypothesis that this time window is important to language-related skill development.

We also found a significant correlation between rapid naming ability and auditory late component amplitude. Previous research (Kuuluvainen et al., 2016) showed a similar relationship between N4 and rapid naming speed in preschool children in which N4 was suggested to be linked to accessing phonological representations. Overall, the correlation patterns found in the current study between the phonological and rapid naming ability and auditory brain responses are consistent with and in support of the earlier literature.

Audiovisual responses shared a large portion of variance with the auditory responses, and furthermore, both showed an association with phonology. In order to disentangle contributions of the auditory processing from the audiovisual processing, we run regression analyses with both auditory and audiovisual brain responses as predictors. No unique variance was left to be explained by the responses to the audiovisual stimuli on the phonological skills after the left auditory late response was taken into account. The regression analyses thus showed the auditory response to be the driving force behind the association with phonological skills.

N170 amplitude and the laterality index of the N170 were not significantly correlated with any of the cognitive skills in the visual only condition. Most previous studies (Cohen et al., 2000; Dehaene et al., 2002; Maurer et al., 2005, 2008; Dehaene and Cohen, 2011) found brain specialization for letter strings and whole words in VWFA (as indexed by N170 responses in EEG/MEG). Presentation of single letters in our study instead of letter strings or words could therefore have led to the lack of findings for the N170 response in the visual only condition. However, previous studies (McCandliss and Noble, 2003; Maurer et al., 2010) have suggested the left lateralization of N170 for words to be partly driven by an automatic link between orthographic and phonological systems. Interestingly, the N170 response showed significant correlation with phonological skills in both audiovisual congruent and incongruent conditions in the left fusiform area. This result could suggest a possible top-down feedback activation of the VWFA and the lateral inferior temporal cortex from auditory and audiovisual integration sites. It has been reported that the VWFA could be activated during speech processing through a top-down modulation (Dehaene et al., 2010; Desroches et al., 2010; Yoncheva et al., 2010). Such auditory/audiovisual processing modulation fits well with the significant correlation between phonological processing and N170 responses in left fusiform in the audiovisual conditions in our study. Similar results were found in an MEG study in which occipitotemporal letter-string-sensitive activation strength was also reported to be correlated with phonological skills in children (Parviainen et al., 2006).

When comparing the summed unimodal responses to the audiovisual responses, suppressive audiovisual integration effect was found in right temporal and both left and right parietal regions. These regions partly match with a previous MEG study (Raij et al., 2000) in adults about LSS integration in which a suppressive integration effect was found in the right temporo-occipito-parietal junction and the left and right STS. In the current study, we found suppressive audiovisual integration effects mostly in the temporoparietal areas but not in the frontal cortices reported in (Raij et al., 2000). This could be due to the difference in the experimental design since an active implicit audiovisual task was used in our study whereas (Raij et al., 2000) used an active explicit matching task, which could recruit more top-down task related audiovisual attention processes (van Atteveldt et al., 2007). The dorsal (temporoparietal) system, including supramarginal gyrus/angular gyrus in the inferior parietal lobule and the posterior superior temporal gyrus (pSTG) is thought to be related to mapping visual print onto the phonological and semantic structures of language (Sandak et al., 2004). Compared with the rather consistent findings in the superior temporal cortex for LSS integration in adults (Raij et al., 2000; van Atteveldt et al., 2004), it seems that the early readers have recruited more widely distributed temporoparietal cortical networks to support learning the association of orthography with phonological codes (Pugh et al., 2013). The suppressive LSS integration effect in the parietal areas at the rather late time window could be related to top-down modulation of the audiovisual processing and reflect less automatic processing of the stimuli than in adults. Pugh et al. (2013) also find a similar correlation between BOLD response and reading skills in the precuneus, which is similar to the current study, and they interpret their finding as part of the visual attention network that seems to impact reading development. They also suggest that this could reflect the integration between visual, language and attentional processes. Lack of the suppressive integration effect at the left superior temporal areas could be related to the less automatic processing of the multimodal stimuli in early readers (Froyen et al., 2009; Blomert, 2011).

The timing of this integration effect was mostly from about 300 to 600 ms in the present study, which matches well with the previous studies using similar stimuli and paradigms (Raij et al., 2000; Herdman et al., 2006; Jost et al., 2014). The relatively late time window is probably due to the fact that bimodal audiovisual integration happens after the early unimodal processing of sound in the auditory cortex and print in the visual cortex (Raij et al., 2000) and possibly involve the feedback projection to auditory cortex in a late stage of processing (van Atteveldt et al., 2004).

Significant partial correlations were found between the audiovisual integration effect and phonological skills, rapid naming abilities as well as reading and writing skills. Phonological skills were correlated with the strength of the audiovisual integration effect in the right inferior parietal and precuneus regions, while rapid naming of letters was correlated with the strength of the audiovisual integration in the left supramarginal and right precuneus regions. Previously research has found similar associations between both structural (gray matter volume indices) (Raschle et al., 2011) and functional (Raschle et al., 2012) changes in these temporoparietal regions and pre-reading skills such as phonology and rapid naming. Moreover, activations in left parietal (angular gyrus) lobe was correlated with individual at-risk index scores for dyslexia in pre-readers (Specht et al., 2009). Reduced LSS is suggested to be linked to a deficit in auditory processing of speech sounds, which in turn predicts phonological skills (Blau et al., 2009). Consistent correlation was found between the strength of the audiovisual integration effect in the right precuneus and reading skills such as word list, nonword list and nonword text reading accuracy. This matches well with results from one recent study which used similar brain-behavior correlation analysis with fMRI and showed the activation in the precuneus to print and speech sounds of words and pseudowords to be correlated with reading-related skills (Pugh et al., 2013). Finally, writing skills were also significantly correlated with the strength of the audiovisual integration effect in the right superior and middle temporal regions. This might suggest that the skills required in writing to dictation are more associated with auditory processes for speech than those required for reading (Hämäläinen et al., 2009). Taken together, these results highlight the important role of LSS in the temporoparietal area in early reading acquisition (Blomert and Froyen, 2010; Blomert and Willems, 2010).

Audio-visual congruency did not produce significant effects in the brain responses in the present study. Here we discuss possible reasons for this. First, the congruency effect which heavily depends on the task demands (Andersen et al., 2004; van Atteveldt et al., 2007), also seems to interact with the brain imaging method (fMRI vs. MEG). For example, several previous fMRI studies on children have found a congruency effect using similar implicit active tasks to ours (Blau et al., 2010; Brem et al., 2010). In contrast, use of an active explicit matching task in fMRI has been reported to overrule the congruency effect (van Atteveldt et al., 2007) However, the MEG study of (Raij et al., 2000) used an active task forcing the participants to relate letters to sounds and reported an audiovisual congruency effect in the heteromodal superior temporal cortex. Therefore, it seems that the task demands modulate differently the MEG and BOLD responses. Second, it is also possible that the children in the present study may not establish fully automatized LSS integration as many of them only have 1 or 2 years of reading instruction. Previous research (Froyen et al., 2009; Blomert, 2011) using MMN paradigm has shown the protracted developmental trajectory of LSS integration and this may be reflected in the absence of congruency effect in the present study. Finally, almost all previous electrophysiological studies (Froyen et al., 2008, 2010; Žarić et al., 2014) examining letter-speech sound congruency in children have used an oddball paradigm, it is likely that congruency is pronounced in the oddball paradigm, but not in the simple LSS paradigm used in the present study. The audiovisual integration and congruency comparisons indicated that children seemed to utilize more general multimodal integration processes of letters and speech sounds, but have not reached the fully automatic level of integration as shown by the absent of congruency effect.

A cohort of beginning readers with relatively wide age range (6–11 years) was recruited to examine the reading and reading related cognitive skills as continuums. Even though we controlled for age in all of the correlation and regression analyses, age did not seem to have a large impact on the results. This finding is similar to that of, for example, the study by Pugh et al. (2013). It seems that the correlations were driven more by learning of these cognitive skills than general maturation of the central nervous system.

According to the general neurodevelopmental theory for reading proposed by (Pugh et al., 2000a; Cornelissen et al., 2010), the temporal and dorsal parietal networks are crucial for the early stage of reading acquisition. Working together with the anterior regions (especially the inferior frontal gyrus), the dorsal (temporoparietal) reading system is involved in the emergence of phonological awareness (Katzir et al., 2005) and in forming associations between orthography, phonology, and semantics (Pugh et al., 2001). Such associations will then shape the organization and connectivity of left occipitotemporal regions including the VWFA (Dehaene and Cohen, 2011) for supporting fluent reading in advanced readers. The present study highlighted the important role of the temporoparietal route in developing phonological awareness and forming automatic LSS in early readers.

A possible concern regarding our study relates to the accuracy of MEG source reconstruction in children, which could be affected by many factors including the relatively large distance of the child’s head to the MEG sensors, imprecise cortical surface reconstruction, suboptimal forward and inverse solution parameters for the child brain and potential MEG-MRI coregistration errors. These could lead to misallocation of brain activity to neighboring brain regions from their true locations in the source analyses. In general, we followed the recommended analysis practice proposed by (Jas et al., 2017) and checked in each step the quality of the data carefully. Furthermore, MEG is less sensitive to the conductivity parameters of the head tissues than EEG which should allow better reconstruction of source activity in children. In addition, we used relatively large brain regions, and in the case of LSS integration effects whole brain analysis, capturing most of the brain activity in the different conditions taking into account possible limitations in localization accuracy of the brain activity.

## Conclusion

In conclusion, brain-behavior analyses were used to explore the relationship between behavioral tasks measuring different cognitive skills and brain responses related to auditory and visual processing of letters and speech sounds in beginning readers. Regression analysis identified the auditory late component in response to speech sounds to be the most significant predictor of phonological skills and rapid naming. In addition, the audiovisual integration effect was found in left and right temporoparietal regions and several of these temporal and parietal regions showed contribution to reading and writing skills. Findings from the current study point to the important role of temporoparietal regions in learning letter-speech sound associations in early reading development. A more detailed neurocognitive model, including additional measures such as functional connectivity, is needed for better understanding of the cortical organization and the developmental trajectory of LSS in children learning to read.

## Author Contributions

WX, JH, and OK designed the study. WX, JH, and OK performed the MEG experiments. WX, JH, and SM analyzed the data. All authors discussed the results and contributed to the final manuscript.

## Conflict of Interest Statement

The authors declare that the research was conducted in the absence of any commercial or financial relationships that could be construed as a potential conflict of interest.

## References

[B1] Albrecht R. Suchodoletz W. Uwer R. (2000). The development of auditory evoked dipole source activity from childhood to adulthood. *Clin. Neurophysiol.* 111 2268–2276. 10.1016/S1388-2457(00)00464-8 11090781

[B2] Andersen T. S. Tiippana K. Sams M. (2004). Factors influencing audiovisual fission and fusion illusions. *Brain Res. Cogn. Brain Res.* 21 301–308. 10.1016/j.cogbrainres.2004.06.004 15511646

[B3] Bach S. Richardson U. Brandeis D. Martin E. Brem S. (2013). Print-specific multimodal brain activation in kindergarten improves prediction of reading skills in second grade. *Neuroimage* 82 605–615. 10.1016/j.neuroimage.2013.05.062 23727320

[B4] Bann S. A. Herdman A. T. (2016). Event related potentials reveal early phonological and orthographic processing of single letters in letter-detection and letter-rhyme paradigms. *Front. Hum. Neurosci.* 10:176. 10.3389/fnhum.2016.00176 27148023 PMC4840210

[B5] Besle J. Fischer C. Bidet-Caulet A. Lecaignard F. Bertrand O. Giard M.-H. (2008). Visual activation and audiovisual interactions in the auditory cortex during speech perception: intracranial recordings in humans. *J. Neurosci.* 28 14301–14310. 10.1523/JNEUROSCI.2875-08.2008 19109511 PMC6671467

[B6] Besle J. Fort A. Delpuech C. Giard M.-H. (2004). Bimodal speech: early suppressive visual effects in human auditory cortex. *Eur. J. Neurosci.* 20 2225–2234. 10.1111/j.1460-9568.2004.03670.x 15450102 PMC1885424

[B7] Binder J. R. Desai R. H. Graves W. W. Conant L. L. (2009). Where is the semantic system? A critical review and meta-analysis of 120 functional neuroimaging studies. *Cereb. Cortex* 19 2767–2796. 10.1093/cercor/bhp055 19329570 PMC2774390

[B8] Blau V. Reithler J. Van Atteveldt N. Seitz J. Gerretsen P. Goebel R. (2010). Deviant processing of letters and speech sounds as proximate cause of reading failure: a functional magnetic resonance imaging study of dyslexic children. *Brain* 133 868–879. 10.1093/brain/awp308 20061325

[B9] Blau V. van Atteveldt N. Ekkebus M. Goebel R. Blomert L. (2009). Reduced neural integration of letters and speech sounds links phonological and reading deficits in adult dyslexia. *Curr. Biol.* 19 503–508. 10.1016/j.cub.2009.01.065 19285401

[B10] Blau V. van Atteveldt N. Formisano E. Goebel R. Blomert L. (2008). Task-irrelevant visual letters interact with the processing of speech sounds in heteromodal and unimodal cortex. *Eur. J. Neurosci.* 28 500–509. 10.1111/j.1460-9568.2008.06350.x 18702722

[B11] Blomert L. (2011). The neural signature of orthographic-phonological binding in successful and failing reading development. *Neuroimage* 57 695–703. 10.1016/j.neuroimage.2010.11.003 21056673

[B12] Blomert L. Froyen D. (2010). Multi-sensory learning and learning to read. *Int. J. Psychophysiol.* 77 195–204. 10.1016/j.ijpsycho.2010.06.025 20600371

[B13] Blomert L. Willems G. (2010). Is there a causal link from a phonological awareness deficit to reading failure in children at familial risk for dyslexia? *Dyslexia* 16 300–317. 10.1002/dys.405 20957685

[B14] Bonte M. (2004). Developmental changes in ERP correlates of spoken word recognition during early school years: a phonological priming study. *Clin. Neurophysiol.* 115 409–423. 10.1016/s1388-2457(03)00361-4 14744584

[B15] Bonte M. L. Blomert L. (2004). Developmental dyslexia: ERP correlates of anomalous phonological processing during spoken word recognition. *Brain Res. Cogn. Brain Res.* 21 360–376. 10.1016/j.cogbrainres.2004.06.010 15511652

[B16] Booth J. R. Burman D. D. Meyer J. R. Gitelman D. R. Parrish T. B. Mesulam M. M. (2003). Relation between brain activation and lexical performance. *Hum. Brain Mapp.* 19 155–169. 10.1002/hbm.10111 12811732 PMC6871810

[B17] Brandwein A. B. Foxe J. J. Russo N. N. Altschuler T. S. Gomes H. Molholm S. (2011). The development of audiovisual multisensory integration across childhood and early adolescence: a high-density electrical mapping study. *Cereb. Cortex* 21 1042–1055. 10.1093/cercor/bhq170 20847153 PMC3077428

[B18] Brem S. Bach S. Kucian K. Guttorm T. K. Martin E. Lyytinen H. (2010). Brain sensitivity to print emerges when children learn letter-speech sound correspondences. *Proc. Natl. Acad. Sci. U.S.A.* 107 7939–7944. 10.1073/pnas.0904402107 20395549 PMC2867899

[B19] Buchsbaum B. R. D’Esposito M. (2008). The search for the phonological store: from loop to convolution. *J. Cogn. Neurosci.* 20 762–778. 10.1162/jocn.2008.20501 18201133

[B20] Calvert G. A. Thesen T. (2004). Multisensory integration: methodological approaches and emerging principles in the human brain. *J. Physiol. Paris* 98 191–205. 10.1016/j.jphysparis.2004.03.018 15477032

[B21] Ceponiene R. Alku P. Westerfield M. Torki M. Townsend J. (2005). ERPs differentiate syllable and nonphonetic sound processing in children and adults. *Psychophysiology* 42 391–406. 10.1111/j.1469-8986.2005.00305.x 16008768

[B22] Ceponiene R. Shestakova A. Balan P. Alku P. Yiaguchi K. Naatanen R. (2001). Children’s auditory event-related potentials index sound complexity and “speechness.” *Int. J. Neurosci.* 109 245–260. 10.3109/00207450108986536 11699331

[B23] Ceponiene R. Torki M. Alku P. Koyama A. Townsend J. (2008). Event-related potentials reflect spectral differences in speech and non-speech stimuli in children and adults. *Clin. Neurophysiol.* 119 1560–1577. 10.1016/j.clinph.2008.03.005 18456550 PMC2444016

[B24] Cohen L. Dehaene S. Naccache L. Lehéricy S. Dehaene-Lambertz G. Hénaff M. A. (2000). The visual word form area: spatial and temporal characterization of an initial stage of reading in normal subjects and posterior split-brain patients. *Brain* 123(Pt 2), 291–307. 10.1093/brain/123.2.291 10648437

[B25] Cornelissen P. Hansen P. Kringelbach M. Pugh K. (2010). *The Neural Basis of Reading.* Oxford: Oxford University Press 10.1093/acprof:oso/9780195300369.001.0001

[B26] Dehaene S. Cohen L. (2011). The unique role of the visual word form area in reading. *Trends Cogn. Sci.* 15 254–262. 10.1016/j.tics.2011.04.003 21592844

[B27] Dehaene S. Cohen L. Morais J. Kolinsky R. (2015). Illiterate to literate: behavioural and cerebral changes induced by reading acquisition. *Nat. Rev. Neurosci.* 16 234–244. 10.1038/nrn3924 25783611

[B28] Dehaene S. Le Clec’H G. Poline J.-B. Le Bihan D. Cohen L. (2002). The visual word form area: a prelexical representation of visual words in the fusiform gyrus. *Neuroreport* 13 321–325. 10.1097/00001756-200203040-00015 11930131

[B29] Dehaene S. Pegado F. Braga L. W. Ventura P. Nunes Filho G. Jobert A. (2010). How learning to read changes the cortical networks for vision and language. *Science* 330 1359–1364. 10.1126/science.1194140 21071632

[B30] Denckla M. B. Rudel R. G. (1976). Rapid “automatized” naming (R.A.N.): dyslexia differentiated from other learning disabilities. *Neuropsychologia* 14 471–479. 10.1016/0028-3932(76)90075-0 995240

[B31] Desikan R. S. Ségonne F. Fischl B. Quinn B. T. Dickerson B. C. Blacker D. (2006). An automated labeling system for subdividing the human cerebral cortex on MRI scans into gyral based regions of interest. *Neuroimage* 31 968–980. 10.1016/j.neuroimage.2006.01.021 16530430

[B32] Desroches A. S. Cone N. E. Bolger D. J. Bitan T. Burman D. D. Booth J. R. (2010). Children with reading difficulties show differences in brain regions associated with orthographic processing during spoken language processing. *Brain Res.* 1356 73–84. 10.1016/j.brainres.2010.07.097 20691675 PMC2942963

[B33] Eklund K. Torppa M. Aro M. Leppänen P. H. T. Lyytinen H. (2015). Literacy skill development of children with familial risk for dyslexia through grades 2, 3, and 8. *J. Educ. Psychol.* 107 126–140. 10.1037/a0037121

[B34] Espy K. A. Molfese D. L. Molfese V. J. Modglin A. (2004). Development of auditory event-related potentials in young children and relations to word-level reading abilities at age 8 years. *Ann. Dyslexia* 54 9–38. 10.1007/s11881-004-0002-3 15765002 PMC2729145

[B35] Fraga González G. Žarić G. Tijms J. Bonte M. Blomert L. Leppänen P. (2016). Responsivity to dyslexia training indexed by the N170 amplitude of the brain potential elicited by word reading. *Brain Cogn.* 106 42–54. 10.1016/j.bandc.2016.05.001 27200495

[B36] Fraga González G. Zaric G. Tijms J. Bonte M. Blomert L. van der Molen M. W. (2014). Brain-potential analysis of visual word recognition in dyslexics and typically reading children. *Front. Hum. Neurosci.* 8:474. 10.3389/fnhum.2014.00474 25071507 PMC4075352

[B37] Fraga González G. Žarić G. Tijms J. Bonte M. van der Molen M. W. (2017). Contributions of letter-speech sound learning and visual print tuning to reading improvement: evidence from brain potential and dyslexia training studies. *Brain Sci.* 7:E10. 10.3390/brainsci7010010 28106790 PMC5297299

[B38] Froyen D. van Atteveldt N. Blomert L. (2010). Exploring the role of low level visual processing in letter-speech sound integration: a visual MMN study. *Front. Integr. Neurosci.* 4:9. 10.3389/fnint.2010.00009 20428501 PMC2859813

[B39] Froyen D. Van Atteveldt N. Bonte M. Blomert L. (2008). Cross-modal enhancement of the MMN to speech-sounds indicates early and automatic integration of letters and speech-sounds. *Neurosci. Lett.* 430 23–28. 10.1016/j.neulet.2007.10.014 18023979

[B40] Froyen D. J. W. Bonte M. L. van Atteveldt N. Blomert L. (2009). The long road to automation: neurocognitive development of letter-speech sound processing. *J. Cogn. Neurosci.* 21 567–580. 10.1162/jocn.2009.21061 18593266

[B41] Gramfort A. Luessi M. Larson E. Engemann D. A. Strohmeier D. Brodbeck C. (2013). MEG and EEG data analysis with MNE-Python. *Front. Neurosci.* 7:267. 10.3389/fnins.2013.00267 24431986 PMC3872725

[B42] Groppe D. M. Urbach T. P. Kutas M. (2011). Mass univariate analysis of event-related brain potentials/fields I: a critical tutorial review. *Psychophysiology* 48 1711–1725. 10.1111/j.1469-8986.2011.01273.x 21895683 PMC4060794

[B43] Hämäläinen J. A. Guttorm T. K. Richardson U. Alku P. Lyytinen H. Leppänen P. H. T. (2013). Auditory event-related potentials measured in kindergarten predict later reading problems at school age. *Dev. Neuropsychol.* 38 550–566. 10.1080/87565641.2012.718817 24219695

[B44] Hämäläinen J. A. Leppänen P. H. T. Eklund K. Thomson J. Richardson U. Guttorm T. K. (2009). Common variance in amplitude envelope perception tasks and their impact on phoneme duration perception and reading and spelling in Finnish children with reading disabilities. *Appl. Psycholinguist.* 30 511–530. 10.1017/s0142716409090250

[B45] Hämäläinen J. A. Lohvansuu K. Ervast L. Leppänen P. H. T. (2015). Event-related potentials to tones show differences between children with multiple risk factors for dyslexia and control children before the onset of formal reading instruction. *Int. J. Psychophysiol.* 95 101–112. 10.1016/j.ijpsycho.2014.04.004 24746550

[B46] Hämäläinen M. S. Ilmoniemi R. J. (1994). Interpreting magnetic fields of the brain: minimum norm estimates. *Med. Biol. Eng. Comput.* 32 35–42. 10.1007/BF02512476 8182960

[B47] Hardy M. Smythe P. C. Stennett R. G. (1972). Developmental patterns in elemental reading skills: phoneme-grapheme and grapheme-phoneme correspondences. *J. Educ. Psychol.* 63 433–436. 10.1037/h0033240 5075483

[B48] Häyrinen T. Serenius-Sirve S. Korkman M. (1999). *Lukilasse. Lukemisen, Kirjoittamisen Ja Laskemisen Seulontatestistö Peruskoulun Ala-Asteen Luokille.* Helsinki: Psykologien Kustannus Oy, 1–6.

[B49] Hein G. Doehrmann O. Muller N. G. Kaiser J. Muckli L. Naumer M. J. (2007). Object familiarity and semantic congruency modulate responses in cortical audiovisual integration areas. *J. Neurosci.* 27 7881–7887. 10.1523/JNEUROSCI.1740-07.2007 17652579 PMC6672730

[B50] Herdman A. T. Fujioka T. Chau W. Ross B. Pantev C. Picton T. W. (2006). Cortical oscillations related to processing congruent and incongruent grapheme–phoneme pairs. *Neurosci. Lett.* 399 61–66. 10.1016/j.neulet.2006.01.069 16507333

[B51] Hyvärinen A. Oja E. (2000). Independent component analysis: algorithms and applications. *Neural Netw.* 13 411–430. 10.1016/s0893-6080(00)00026-5 10946390

[B52] Jas M. Larson E. Engemann D.-A. Leppakangas J. Taulu S. Hamalainen M. (2017). MEG/EEG group study with MNE: recommendations, quality assessments and best practices. *bioRxiv* [Preprint]. 10.1101/240044 PMC6088222 30127712

[B53] Jones J. A. Callan D. E. (2003). Brain activity during audiovisual speech perception: an fMRI study of the McGurk effect. *Neuroreport* 14 1129–1133. 10.1097/01.wnr.0000074343.81633.2a 12821795

[B54] Jost L. B. Eberhard-Moscicka A. K. Frisch C. Dellwo V. Maurer U. (2014). Integration of spoken and written words in beginning readers: a topographic ERP study. *Brain Topogr.* 27 786–800. 10.1007/s10548-013-0336-4 24271979

[B55] Katzir T. Misra M. Poldrack R. A. (2005). Imaging phonology without print: assessing the neural correlates of phonemic awareness using fMRI. *Neuroimage* 27 106–115. 10.1016/j.neuroimage.2005.04.013 15901490

[B56] Korkman M. Kirk U. Kemp S. (2007). *NEPSY (NEPSY-II)*, 2nd Edn San Antonio, TX: Harcourt Assessment.

[B57] Kushnerenko E. Ceponiene R. Balan P. Fellman V. Huotilaine M. Näätäne R. (2002). Maturation of the auditory event-related potentials during the first year of life. *Neuroreport* 13 47–51. 10.1097/00001756-200201210-00014 11924892

[B58] Kuuluvainen S. Leminen A. Kujala T. (2016). Auditory evoked potentials to speech and nonspeech stimuli are associated with verbal skills in preschoolers. *Dev. Cogn. Neurosci.* 19 223–232. 10.1016/j.dcn.2016.04.001 27131343 PMC6988591

[B59] Lin F. H. Witzel T. Ahlfors S. P. Stufflebeam S. M. Belliveau J. W. Hämäläinen M. S. (2006). Assessing and improving the spatial accuracy in MEG source localization by depth-weighted minimum-norm estimates. *Neuroimage* 31 160–171. 10.1016/j.neuroimage.2005.11.054 16520063

[B60] Lohvansuu K. Hämäläinen J. A. Ervast L. Lyytinen H. Leppänen P. H. T. (2018). Longitudinal interactions between brain and cognitive measures on reading development from 6 months to 14 years. *Neuropsychologia* 108 6–12. 10.1016/j.neuropsychologia.2017.11.018 29157996

[B61] Lohvansuu K. Hämäläinen J. A. Tanskanen A. Ervast L. Heikkinen E. Lyytinen H. (2014). Enhancement of brain event-related potentials to speech sounds is associated with compensated reading skills in dyslexic children with familial risk for dyslexia. *Int. J. Psychophysiol.* 94 298–310. 10.1016/j.ijpsycho.2014.10.002 25312203

[B62] Lovio R. Näätänen R. Kujala T. (2010). Abnormal pattern of cortical speech feature discrimination in 6-year-old children at risk for dyslexia. *Brain Res.* 1335 53–62. 10.1016/j.brainres.2010.03.097 20381471

[B63] Lyytinen H. Erskine J. Kujala J. Ojanen E. Richardson U. (2009). In search of a science-based application: a learning tool for reading acquisition. *Scand. J. Psychol.* 50 668–675. 10.1111/j.1467-9450.2009.00791.x 19930268

[B64] Maris E. Oostenveld R. (2007). Nonparametric statistical testing of EEG- and MEG-data. *J. Neurosci. Methods* 164 177–190. 10.1016/j.jneumeth.2007.03.024 17517438

[B65] Maurer U. Blau V. C. Yoncheva Y. N. McCandliss B. D. (2010). Development of visual expertise for reading: rapid emergence of visual familiarity for an artificial script. *Dev. Neuropsychol.* 35 404–422. 10.1080/87565641.2010.480916 20614357 PMC3008655

[B66] Maurer U. Brem S. Bucher K. Brandeis D. (2005). Emerging neurophysiological specialization for letter strings. *J. Cogn. Neurosci.* 17 1532–1552. 10.1162/089892905774597218 16269095

[B67] Maurer U. Brem S. Kranz F. Bucher K. Benz R. Halder P. (2006). Coarse neural tuning for print peaks when children learn to read. *Neuroimage* 33 749–758. 10.1016/j.neuroimage.2006.06.025 16920367

[B68] Maurer U. Zevin J. D. McCandliss B. D. (2008). Left-lateralized N170 effects of visual expertise in reading: evidence from Japanese syllabic and logographic scripts. *J. Cogn. Neurosci.* 20 1878–1891. 10.1162/jocn.2008.20125 18370600 PMC4416222

[B69] McCandliss B. D. Cohen L. Dehaene S. (2003). The visual word form area: expertise for reading in the fusiform gyrus. *Trends Cogn. Sci.* 7 293–299. 10.1016/S1364-6613(03)00134-7 12860187

[B70] McCandliss B. D. Noble K. G. (2003). The development of reading impairment: a cognitive neuroscience model. *Ment. Retard. Dev. Disabil. Res. Rev.* 9 196–204. 10.1002/mrdd.10080 12953299

[B71] Melby-Lervåg M. Lyster S.-A. H. Hulme C. (2012). Phonological skills and their role in learning to read: a meta-analytic review. *Psychol. Bull.* 138 322–352. 10.1037/a0026744 22250824

[B72] Näätänen R. (2001). The perception of speech sounds by the human brain as reflected by the mismatch negativity (MMN) and its magnetic equivalent (MMNm). *Psychophysiology* 38 1–21. 10.1111/1469-8986.3810001 11321610

[B73] Ojanen V. Möttönen R. Pekkola J. Jääskeläinen I. P. Joensuu R. Autti T. (2005). Processing of audiovisual speech in Broca’s area. *Neuroimage* 25 333–338. 10.1016/j.neuroimage.2004.12.001 15784412

[B74] Parviainen T. Helenius P. Poskiparta E. Niemi P. Salmelin R. (2006). Cortical sequence of word perception in beginning readers. *J. Neurosci.* 26 6052–6061. 10.1523/JNEUROSCI.0673-06.2006 16738248 PMC6675213

[B75] Parviainen T. Helenius P. Poskiparta E. Niemi P. Salmelin R. (2011). Speech perception in the child brain: cortical timing and its relevance to literacy acquisition. *Hum. Brain Mapp.* 32 2193–2206. 10.1002/hbm.21181 21391257 PMC6870499

[B76] Parviainen T. Helenius P. Salmelin R. (2005). Cortical differentiation of speech and nonspeech sounds at 100 ms: implications for dyslexia. *Cereb. Cortex* 15 1054–1063. 10.1093/cercor/bhh206 15563727

[B77] Pennington B. F. Lefly D. L. (2001). Early reading development in children at family risk for dyslexia. *Child Dev.* 72 816–833. 10.1111/1467-8624.00317 11405584

[B78] Ponton C. W. Eggermont J. J. Kwong B. Don M. (2000). Maturation of human central auditory system activity: evidence from multi-channel evoked potentials. *Clin. Neurophysiol.* 111 220–236. 10.1016/S1388-2457(99)00236-9 10680557

[B79] Preston J. L. Molfese P. J. Frost S. J. Mencl W. E. Fulbright R. K. Hoeft F. (2016). Print-speech convergence predicts future reading outcomes in early readers. *Psychol. Sci.* 27 75–84. 10.1177/0956797615611921 26589242 PMC4713346

[B80] Price C. J. (2000). The anatomy of language: contributions from functional neuroimaging. *J. Anat.* 197 335–359. 10.1046/j.1469-7580.2000.19730335.x 11117622 PMC1468137

[B81] Pugh K. R. Landi N. Preston J. L. Mencl W. E. Austin A. C. Sibley D. (2013). The relationship between phonological and auditory processing and brain organization in beginning readers. *Brain Lang.* 125 173–183. 10.1016/j.bandl.2012.04.004 22572517 PMC3417084

[B82] Pugh K. R. Mencl W. E. Jenner A. R. Katz L. Frost S. J. Lee J. R. (2000a). Functional neuroimaging studies of reading and reading disability (developmental dyslexia). *Ment. Retard. Dev. Disabil. Res. Rev.* 6 207–213. 10.1002/1098-2779(2000)6:3<207::AID-MRDD8>3.0.CO;2-P 10982498

[B83] Pugh K. R. Mencl W. E. Shaywitz B. A. Shaywitz S. E. Fulbright R. K. Constable R. T. (2000b). The angular gyrus in developmental dyslexia: task-specific differences in functional connectivity within posterior cortex. *Psychol. Sci.* 11 51–56. 10.1111/1467-9280.00214 11228843

[B84] Pugh K. R. Mencl W. E. Jenner A. R. Katz L. Frost S. J. Lee J. R. (2001). Neurobiological studies of reading and reading disability. *J. Commun. Disord.* 34 479–492. 10.1016/S0021-9924(01)00060-0 11725860

[B85] Puolakanaho A. Ahonen T. Aro M. Eklund K. Leppänen P. H. T. Poikkeus A.-M. (2007). Very early phonological and language skills: estimating individual risk of reading disability. *J. Child Psychol. Psychiatry* 48 923–931. 10.1111/j.1469-7610.2007.01763.x 17714377

[B86] Raij T. Uutela K. Hari R. (2000). Audiovisual integration of letters in the human brain. *Neuron* 28 617–625. 10.1016/S0896-6273(00)00138-0 11144369

[B87] Raschle N. M. Chang M. Gaab N. (2011). Structural brain alterations associated with dyslexia predate reading onset. *Neuroimage* 57 742–749. 10.1016/j.neuroimage.2010.09.055 20884362 PMC3499031

[B88] Raschle N. M. Zuk J. Gaab N. (2012). Functional characteristics of developmental dyslexia in left-hemispheric posterior brain regions predate reading onset. *Proc. Natl. Acad. Sci. U.S.A.* 109 2156–2161. 10.1073/pnas.1107721109 22308323 PMC3277560

[B89] Rueckl J. G. Paz-Alonso P. M. Molfese P. J. Kuo W.-J. Bick A. Frost S. J. (2015). Universal brain signature of proficient reading: evidence from four contrasting languages. *Proc. Natl. Acad. Sci. U.S.A.* 112 15510–15515. 10.1073/pnas.1509321112 26621710 PMC4687557

[B90] Rüsseler J. Ye Z. Gerth I. Szycik G. R. Münte T. F. (2017). Audio-visual speech perception in adult readers with dyslexia: an fMRI study. *Brain Imaging Behav.* 12 357–368. 10.1007/s11682-017-9694-y 28290075

[B91] Sandak R. Einar Mencl W. Frost S. J. Pugh K. R. (2004). The neurobiological basis of skilled and impaired reading: recent findings and new directions. *Sci. Stud. Read.* 8 273–292. 10.1207/s1532799xssr0803_6

[B92] Schlaggar B. L. McCandliss B. D. (2007). Development of neural systems for reading. *Annu. Rev. Neurosci.* 30 475–503. 10.1146/annurev.neuro.28.061604.135645 17600524

[B93] Shankweiler D. Mencl W. E. Braze D. Tabor W. Pugh K. R. Fulbright R. K. (2008). Reading differences and brain: cortical integration of speech and print in sentence processing varies with reader skill. *Dev. Neuropsychol.* 33 745–775. 10.1080/87565640802418688 19005913

[B94] Sliwinska M. W. James A. Devlin J. T. (2015). Inferior parietal lobule contributions to visual word recognition. *J. Cogn. Neurosci.* 27 593–604. 10.1162/jocn_a_00721 25244114

[B95] Specht K. Hugdahl K. Ofte S. Nygård M. Bjørnerud A. Plante E. (2009). Brain activation on pre-reading tasks reveals at-risk status for dyslexia in 6-year-old children. *Scand. J. Psychol.* 50 79–91. 10.1111/j.1467-9450.2008.00688.x 18826418

[B96] Sperdin H. F. Cappe C. Foxe J. J. Murray M. M. (2009). Early, low-level auditory-somatosensory multisensory interactions impact reaction time speed. *Front. Integr. Neurosci.* 3:2. 10.3389/neuro.07.002.2009 19404410 PMC2659167

[B97] Stein B. E. Stanford T. R. (2008). Multisensory integration: current issues from the perspective of the single neuron. *Nat. Rev. Neurosci.* 9 255–266. 10.1038/nrn2331 18354398

[B98] Stevens C. McIlraith A. Rusk N. Niermeyer M. Waller H. (2013). Relative laterality of the N170 to single letter stimuli is predicted by a concurrent neural index of implicit processing of letternames. *Neuropsychologia* 51 667–674. 10.1016/j.neuropsychologia.2012.12.009 23274433

[B99] Szymanski M. D. Rowley H. A. Roberts T. P. (1999). A hemispherically asymmetrical MEG response to vowels. *Neuroreport* 10 2481–2486. 10.1097/00001756-199908200-00009 10574356

[B100] Taulu S. Kajola M. (2005). Presentation of electromagnetic multichannel data: the signal space separation method. *J. Appl. Phys.* 97:124905 10.1063/1.1935742

[B101] Taulu S. Kajola M. Simola J. (2004). Suppression of interference and artifacts by the signal space separation method. *Brain Topogr.* 16 269–275. 10.1023/B:BRAT.0000032864.93890.f9 15379226

[B102] Taulu S. Simola J. (2006). Spatiotemporal signal space separation method for rejecting nearby interference in MEG measurements. *Phys. Med. Biol.* 51 1759–1768. 10.1088/0031-9155/51/7/008 16552102

[B103] Torgesen J. K. Rashotte C. A. Wagner R. K. (1999). *TOWRE: Test of Word Reading Efficiency.* Austin, TX: Pro-Ed.

[B104] Tremblay C. Champoux F. Voss P. Bacon B. A. Lepore F. Théoret H. (2007). Speech and non-speech audio-visual illusions: a developmental study. *PLoS One* 2:e742. 10.1371/journal.pone.0000742 17710142 PMC1937019

[B105] van Atteveldt N. Formisano E. Goebel R. Blomert L. (2004). Integration of letters and speech sounds in the human brain. *Neuron* 43 271–282. 10.1016/j.neuron.2004.06.025 15260962

[B106] van Atteveldt N. Roebroeck A. Goebel R. (2009). Interaction of speech and script in human auditory cortex: insights from neuro-imaging and effective connectivity. *Hear. Res.* 258 152–164. 10.1016/j.heares.2009.05.007 19500658

[B107] van Atteveldt N. M. Formisano E. Goebel R. Blomert L. (2007). Top-down task effects overrule automatic multisensory responses to letter-sound pairs in auditory association cortex. *Neuroimage* 36 1345–1360. 10.1016/j.neuroimage.2007.03.065 17513133

[B108] Vandermosten M. Hoeft F. Norton E. S. (2016). Integrating MRI brain imaging studies of pre-reading children with current theories of developmental dyslexia: a review and quantitative meta-analysis. *Curr. Opin. Behav. Sci.* 10 155–161. 10.1016/j.cobeha.2016.06.007 27458603 PMC4957935

[B109] Weber-Fox C. Spencer R. Cuadrado E. Smith A. (2003). Development of neural processes mediating rhyme judgments: phonological and orthographic interactions. *Dev. Psychobiol.* 43 128–145. 10.1002/dev.10128 12918092

[B110] Wechsler D. (1991). *Manual for the Wechsler Intelligence Scale for Children-(WISC-III).* San Antonio, TX: Psychological Corporation.

[B111] Wechsler D. (2003). *Wechsler Intelligence Scale for Children*, 4th Edn San Antonio, TX: The Psychological Corporation.

[B112] Wunderlich J. L. Cone-Wesson B. K. Shepherd R. (2006). Maturation of the cortical auditory evoked potential in infants and young children. *Hear. Res.* 212 185–202. 10.1016/j.heares.2005.11.010 16459037

[B113] Yoncheva Y. N. Zevin J. D. Maurer U. McCandliss B. D. (2010). Auditory selective attention to speech modulates activity in the visual word form area. *Cereb. Cortex* 20 622–632. 10.1093/cercor/bhp129 19571269 PMC2820701

[B114] Žarić G. González G. F. Tijms J. Van Der Molen M. W. Blomert L. Bonte M. (2014). Reduced neural integration of letters and speech sounds in dyslexic children scales with individual differences in reading fluency. *PLoS One* 9:e110337. 10.1371/journal.pone.0110337 25329388 PMC4199667

