# Supplementary material for: Brain Responses to Letters and Speech Sounds and Their Correlations With Cognitive Skills Related to Reading in Children
Source: Front Hum Neurosci. 2018 Aug 3;12:304. doi: 10.3389/fnhum.2018.00304 (PMC6088176; doi:10.3389/fnhum.2018.00304)
Supplement: Supplementary file 1 [file Table_1.DOCX]

Supplementary Material

Title: Brain Responses to Letters and Speech Sounds and Their Correlations with Cognitive Skills Related to Reading in Children

Weiyong Xu^1,2^ *, Orsolya Beatrix Kolozsvari^1,2^, Simo Petteri Monto^1,2^, and Jarmo Arvid Hämäläinen^1,2^

^1^ Department of Psychology, University of Jyväskylä, Jyväskylä, Finland

^2^ Centre for Interdisciplinary Brain Research, University of Jyväskylä, Jyväskylä, Finland

*** Correspondence:** Weiyong Xu: weiyong.w.xu@jyu.fi

## Supplementary Tables

**Supplementary Table 1** Partial correlations (controlling for age) between cognitive skills and peak latencies of the auditory responses in the auditory only condition. The auditory components are left (L) and right (R) auditory N1m, N2m and late component (LC).

| Auditory | Phonological processing | RAN letters | RAN objects | Word list reading | Non-word list reading | Non-word text time | Non-word text accuracy | Writing to dictation |
| --- | --- | --- | --- | --- | --- | --- | --- | --- |
| L, N1m | -.288 | .245 | .203 | -.153 | -.106 | .120 | -.253 | **-.407*** |
| R, N1m | -.381 | **.406*** | .341 | -.216 | -.184 | .151 | -.032 | .054 |
| L, N2m | .059 | -.209 | -.200 | .193 | .199 | -.297 | -.162 | .092 |
| R, N2m | -.181 | .166 | .045 | -.258 | -.344 | .322 | -.085 | .053 |
| L, LC | -.249 | .357 | -.047 | .053 | .095 | -.314 | -.338 | -.019 |
| R, LC | -.214 | .312 | -.119 | -.171 | -.141 | -.071 | **-.437*** | -.262 |

Note: *p<0.05.

**Supplementary Table 2** Partial correlations (controlling for age) between cognitive skills and peak latencies of the visual responses in the visual only condition. The visual responses are left (L) and right (R) visual P1m in the visual cortices and N170m in the fusiform gyrus.

| Visual | Phonological processing | RAN letters | RAN objects | Word list reading | Non-word list reading | Non-word text time | Non-word text accuracy | Writing to dictation |
| --- | --- | --- | --- | --- | --- | --- | --- | --- |
| L, VC, P1 | .060 | -.118 | .187 | -.062 | -.099 | .009 | .045 | -.237 |
| R, VC, P1 | .306 | **-.394*** | -.376 | **.421*** | .283 | -.357 | .212 | .256 |
| L, FG, N170 | .025 | .037 | -.158 | -.162 | -.114 | .019 | -.081 | -.316 |
| R, FG, N170 | .014 | -.052 | .061 | -.047 | -.106 | .093 | -.167 | -.247 |

Note: VC = visual cortices, FG = fusiform gyrus. *p<0.05.

**Supplementary Table 3** Partial correlations (controlling for age) between cognitive skills and peak latencies of the auditory and visual responses in the audiovisual conditions (the first row in each cell is audiovisual congruent (AVC) and the second row audiovisual incongruent (AVI)). The auditory components are left (L) and right (R) auditory N1m, N2m and late components. The visual components are left (L) and right (R) visual P1m in the visual cortices and N170m in the fusiform gyrus.

| AVC  AVI | Phonological processing | RAN letters | RAN objects | Word list reading | Non-word list reading | Non-word text time | Non-word text accuracy | Writing to dictation |
| --- | --- | --- | --- | --- | --- | --- | --- | --- |
| L, AC, N1 | .060  **-.404*** | .015  .259 | -.073  .053 | -.086  -.182 | -.071  -.129 | -.011  .159 | -.344  -.293 | .000  -.201 |
| R, AC, N1 | .083  -.099 | .061  .177 | .063  .124 | -.118  -.060 | -.074  -.056 | -.024  .032 | .119  .166 | .274  .223 |
| L, AC, N2 | -.038  -.143 | -.141  -.134 | -.228  -.365 | .189  .228 | .066  .211 | -.276  -.224 | -.240  -.089 | .017  -.101 |
| R, AC, N2 | -.142  -.227 | .061  -.009 | .122  -.053 | -.065  -.101 | .021  -.103 | .041  .143 | .121  .031 | .165  -.218 |
| L, AC, LC | -.130  -.049 | .391  .310 | .177  .103 | -.019  -.210 | .078  -.068 | -.026  -.020 | -.035  -.329 | -.014  -.098 |
| R, AC, LC | -.164  -.072 | .233  .088 | -.061  -.114 | -.022  .004 | .046  .102 | -.064  -.099 | -.306  -.219 | .215  -.100 |
| L, VC, P1 | -.002  -.041 | -.064  .017 | -.024  -.005 | .080  -.039 | .042  -.028 | -.224  -.094 | -.100  .034 | -.133  -.189 |
| R, VC, P1 | -.065  -.038 | -.006  -.019 | .015  -.234 | -.059  -.065 | -.079  .043 | -.038  -.085 | -.114  -.102 | -.048  -.169 |
| L, FG, N170 | -.093  .183 | -.165  -.313 | -.073  -.092 | .060  .180 | -.054  .170 | .017  -.067 | -.146  .089 | .019  .228 |
| R, FG, N170 | .071  .017 | -.020  -.062 | -.162  .012 | .034  .128 | .046  .096 | -.029  -.170 | -.163  -.141 | -.167  -.195 |

Note: AC = auditory cortices, VC = visual cortices, FG = fusiform gyrus. *p<0.05.
